# Accumulation of Heavy Metals and Metalloid in Foodstuffs from Agricultural Soils around Tarkwa Area in Ghana, and Associated Human Health Risks

**DOI:** 10.3390/ijerph120808811

**Published:** 2015-07-28

**Authors:** Nesta Bortey-Sam, Shouta M. M. Nakayama, Osei Akoto, Yoshinori Ikenaka, Julius N. Fobil, Elvis Baidoo, Hazuki Mizukawa, Mayumi Ishizuka

**Affiliations:** 1 Laboratory of Toxicology, Graduate School of Veterinary Medicine, Hokkaido University, Kita 18, Nishi 9, Kita ku, Sapporo 060–0818, Japan; E-Mails: borteysam@yahoo.com (N.B.-S.); shouta-nakayama@vetmed.hokudai.ac.jp (S.M.M.N.); Y_ikenaka@vetmed.hokudai.ac.jp (Y.I.); 2 Department of Chemistry, Kwame Nkrumah University of Science and Technology, Kumasi, Ghana; E-Mails: wofakmann@yahoo.com (O.A.); elvixbaid@yahoo.com (E.B.); 3 Water Research Group, Unit for Environmental Sciences and Management, North-West University, Potchefstroom 2531, South Africa; 4 Department of Biological, Environmental & Occupational Health Sciences, School of Public Health, University of Ghana, P.O. Box LG13, Legon, Ghana; E-Mail: jfobil@ug.edu.gh; 5 Department of Environmental Veterinary Science, Graduate School of Veterinary Medicine, Hokkaido University, Kita 18, Nishi 9, Kita ku, Sapporo 060–0818, Japan; E-Mail: hazuki.mizukawa@vetmed.hokudai.ac.jp

**Keywords:** metals, metalloid, health risk, foodstuff, bioconcentration factor, target hazard quotient

## Abstract

This study was carried out to assess the extent of heavy metals and metalloid accumulation from agricultural soils to foodstuffs (viz, *M. esculenta* (cassava) and *Musa paradisiaca* (plantain)) around thirteen neighboring communities within Tarkwa, Ghana; and to estimate the human health risk associated with consumption of these foodstuffs. Concentrations of As, Cd, Co, Cr, Cu, Ni, Pb, and Zn were measured with an inductively coupled plasma–mass spectrometer and mercury analysis was done using a mercury analyzer. From the results, 30% of cassava samples collected, contained higher concentrations of Pb when compared to Codex Alimentarius Commission standard values. Bioconcentration factor indicated that Ni had higher capacity of absorption into food crops from soil than the other heavy metals. For both children and adults, the target hazard quotient (THQ) of Pb in cassava in communities such as Techiman, Wangarakrom, Samahu, and Tebe (only children) were greater than 1, which is defined as an acceptable risk value. This indicated that residents could be exposed to significant health risks associated with cassava consumption.

## 1. Introduction

There has long been concerns about heavy metals and metalloids pollution because of their stability and non-biodegradability in environmental media as well as their toxicity to plants, animals, and humans [1]. Soil is the primary reservoir for heavy metals in the atmosphere, hydrosphere, and biota, and thus plays a fundamental role in the overall metal cycle in nature [2]. Heavy metals in soil pose potential threats to the environment and can cause human health problems through various absorption pathways such as inhalation, absorption through dermal pores, or ingestion (diet through the soil–food chain) [3].

Vegetables/foodstuffs play important roles in our daily diet as economic crops. However, in Ghana, various human activities such as mining, industrial processing (smelting), automobile exhausts, and applications of organic manure/fertilizers are causing elevated heavy metal concentrations in environmental media and absorption by food crops [4,5,6,7,8]. Vegetables/foodstuffs take up heavy metals and/or metalloids by absorbing them from contaminated soils, as well as from deposits on parts of the crop exposed to air from polluted environments [9]. The main pathway by which plants accumulate heavy metals is through root uptake from soils [10]. Some heavy metals present in the soil solution are adsorbed onto the roots, and then become bound to carboxyl groups of mucilage uronic acid, or directly to the polysaccharides of the rhizoderm cell surface [11]. Once adsorbed onto the rhizoderm roots surface, they may enter the roots passively and follow translocating water streams. Indeed, the highest concentrations of some heavy metals can be found in root apices, where root cells are young and have thin cell walls (with the exception of root cap cells) [12]. Heavy metals ( particularly lead) may enter the roots through ionic channels. The amount of heavy metals that move from soil into plants can be measured by “the transfer factor”; this factor is defined as the ratio that exists between the concentrations of metals in the plant and that in soil. This transfer factor could be different for different plant species and will change as soil physical and chemical properties are altered. Generally, plants having a transfer factor greater than 1 are categorized as hyperaccumulators, whereas those with transfer factor less than 1 are termed as non-accumulators [13].

Heavy metals in leaf and tuber crops as a consequence of soil and atmospheric contamination by mining and related activities showed concentrations of cadmium, zinc and lead were detected in the: leaves of cassava growing near the Enyigba base metal deposit in Nigeria [14]; tubers of cassava in the environs of the Arufu base metal deposit in Nigeria [15]; as well as in the tubers of cassava, sweet potato and yam in other districts in the same country [16]. Concentrations of arsenic and zinc in cassava tubers were also investigated in areas where gold was mined in Obuasi, Ghana [17] and Dunkwa-on-Offin, Ghana [18]. Cassava cultivated in areas affected by mining were found to contain higher concentrations of heavy metals/metalloids when compared with those grown in uncontaminated areas [19].

Chronic intake of heavy metals above their safe threshold in humans and animals have damaging effects and can cause non–carcinogenic hazards such as neurologic impairment, headache, and liver disease [20]. Dietary cadmium intake due to consumption of environmentally contaminated rice and other foods was associated with an increased risk of postmenopausal breast cancer [21]. Acute and chronic exposure to arsenic could also cause numerous human health problems. These include dermal, respiratory, cardiovascular, gastrointestinal, hematological, hepatic, renal, neurological, developmental, reproductive, immunological, genotoxic, mutagenic, and carcinogenic effects [22]. Despite the economic benefits of industry, the negative impacts on humans and the environment may cause additional costs not factored into income projections. Especially for the ubiquitous and non-biodegradable heavy metals, the negative effects persist for several decades and even longer [23].

Tarkwa is a noted centre for gold mining, and has a large open–cast gold mine, situated northwest of the town. Tarkwa has nearly a century of gold mining history and the largest concentration of mining companies in a single district in Ghana and the West African sub–region [24]. Studies conducted on the impact of gold mining in soil, drinking water, and foods collected around mining communities in Tarkwa showed high levels of some toxic metals including arsenic and mercury [5,6,25,26,27]. Despite the wide and numerous studies of toxic metal concentrations in various environmental and biological samples in Ghana, there is limited or no data from literature that addresses the accumulation of heavy metals and metalloids in foodstuffs from soil and the potential health risk to humans through consumption.

The objectives of the present study were therefore to assess the extent of heavy metal and metalloid accumulation from soils to foodstuffs commonly grown in agricultural areas in Tarkwa; study the relation between bioconcentration factor (BCF), soil pH and soil organic matter (SOM); and evaluate the potential human health risks of toxic metal exposure to residents in Tarkwa through consumption of foodstuffs.

## 2. Materials and Methods

### 2.1. Study Area

Tarkwa, 05°18′00″N; 01°59′00″W, is the town of interest in this study and is in the southwest of Ghana, located about 120 miles west of the capital city, Accra. As of 2010, Tarkwa was estimated to have a population of 90,477 [28]. Topography of Tarkwa, shows plateaus with heights between 600 and 1050 meters, and south of the town lies a vast stretch of terrain which is suitable for agricultural crop production. Large plantations of orange, rubber, and palm are therefore a major feature of this area. Tarkwa and its environs lie within the transition zone between the tropical rain forest and the coastal shrub vegetation. In Tarkwa, the average rainfall is 850 mm per year, and the maxima is recorded between June and October. Planting and harvesting of food crops are mostly done during this period and cassava, plantain, yam, cocoyam, oranges, and vegetables are the staples grown in this area [25].

### 2.2. Soil, Foodstuff Sampling and Processing

**Figure 1:**
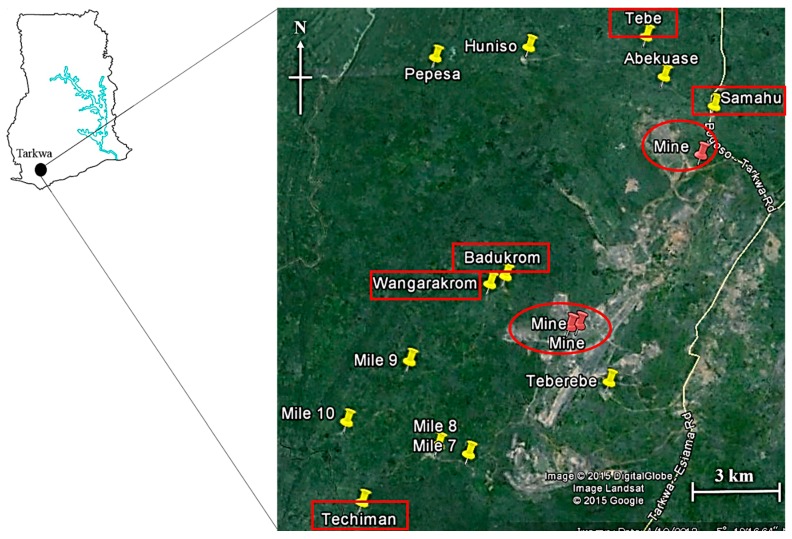
Map showing agricultural soils and foodstuffs sampling locations in Tarkwa (Yellow pins indicate sampled communities and red pins indicate gold mines).

In June, 2012, a total of 60 agricultural soils and 65 foodstuffs were randomly collected from 13 communities in Tarkwa. Global positioning system (GPS) was used to geo-reference the sampling positions (Figure 1). 

Soil samples (0–10 cm top layer) were collected using a stainless steel, and stored in labeled corning tubes (Corning Incorporated, New York, NY, USA). Three to eight agricultural soils were collected from different farms in some communities. Two kinds of foodstuffs viz, *M. esculenta* (cassava) and *Musa paradisiaca* (plantain) were collected during this period. In Ghana, cassava and plantain are important components of human diet and constitute the staples in the region. Cassava and plantain are grown and consumed in many places, particularly in the rural areas of Ghana. In some communities, foodstuffs were collected from different farms and stored in polyethylene zipbags. The samples obtained were stored at –20 ^o^C in the Department of Chemistry, KNUST, Ghana and later transported to the Laboratory of Toxicology, Graduate School of Veterinary Medicine, Hokkaido University, Japan where they were stored at –30 ^o^C until analysis. A map of the sampling area and points is presented in Figure 1.

Prior to chemical analysis, the soil samples were air dried at room temperature and passed through a 2 mm sieve. Soil pH was measured in a soil deionized water suspension (soil: water, 1:2.5 by volume) by a calibrated pH meter. The water content of each soil sample was measured after 12 h of drying in an oven at 105 ^o^C. Soil organic matter (SOM) content was determined by loss of weight on ignition at oven temperature of 600 ^o^C for 5 h. For foodstuff samples, the edible parts were rinsed with deionized water and dried at 45 ^o^C.

### 2.3. Heavy Metal Analyses

Digestion of heavy metals and metalloid in soil samples was done according to method described by Bortey-Sam *et al*. [29]. Briefly, approximately 0.5 g of soil sample was weighed into prewashed digestion vessel and digested (Speedwave two, Berghof, Germany) using 10 mL of 60% HNO_3_ (Kanto Chemical Corporation, Tokyo, Japan). After cooling, samples were filtered into corning tubes (Corning Incorporated, New York, NY, USA) using ashless filter paper 5B (Advantec, Tokyo, Japan). The solution was standardized to 50 mL using distilled, deionised water. Similarly, 0.5 g of foodstuff was weighed and digested in a mixture of HNO_3_:H_2_O_2_ (5:1, *v/v*; 60%:35%) (Kanto Chemical Corporation, Tokyo, Japan) and the solution was made to 10 mL using distilled, deionised water. Blanks were prepared using the same procedure.

Concentrations of arsenic (As), cadmium (Cd), cobalt (Co), chromium (Cr), copper (Cu), nickel (Ni), lead (Pb), and zinc (Zn) were measured with an inductively coupled plasma–mass spectrometer (ICP–MS; 7700 series, Agilent technologies, Tokyo, Japan) and results were expressed in mg/kg dry weight (dw). The concentration of total mercury (Hg) was measured by thermal decomposition, gold amalgamation, and atomic absorption spectrophotometry (Mercury analyzer, MA–3000; Nippon Instruments Corporation, Tokyo, Japan), after preparation of the calibration standards. 

### 2.4. Quality Control and Quality Assurance

For quality control, blanks and duplicates (samples) were analyzed after every 10 sample analyses. The instrument was calibrated using standard solutions of the respective metals (to establish standard curves before metal analysis). The correlation coefficients (*r^2^*) of the calibration curves were greater than 0.999. All chemicals and standard stock solutions were of analytical–reagent grade (Wako Pure Chemicals Industries, Ltd., Osaka, Japan). The detection limits (ng/g) of the heavy metals were 0.002 (As), 0.001 (Cd), 0.0004 (Co), 0.003 (Cr), 0.007 (Cu), 0.003 (Ni), 0.001 (Pb), and 0.046 (Zn), respectively.

For metals, reference materials SRM 1944 (New York/ New Jersey Waterway Sediment) and BCR–320 (Channel Sediment, IRMM, Belgium) were used for method validation. Replicate analysis of these reference materials showed good accuracy and recovery rates ranged from 80–115%. The detection limit of Hg in samples was 2.0 pg total Hg and recovery rates (%) for the three certified reference materials (BCR–320R, SRM 1944, and DOLT–4) ranged from 92–103.

Foodstuff samples were analyzed three times by the ICP–MS and Hg analyzer and the Relative Standard Deviations (RSD) were ≤ 5%.

### 2.5. Data Analyses

#### 2.5.1. Bioconcentration Factor (BCF)

Recently, the application of enrichment factor in the form of transfer factor, BCF, and plant uptake factor have been expanded to research in soil, water system, sediment, as well as assessment of heavy metal pollution in environmental geochemistry [30]. In soil research, BCF is defined as the ratio of the content of a particular element in plant to that in soil (mg/kg dw; average water contents in foodstuffs were 58% (cassava) and 62% (plantain) respectively). BCF is an important quantitative indicator of crop contamination and has commonly been used for estimating metal transfer from soil to plants [31]. BCF-based research showed that the extent of metal enrichment in vegetables is highest in leaf vegetables, followed by tubers and fruit vegetables [32]. Regarding metal concentrations, Cd and Pb commonly occur at high levels in leafy vegetables, while Zn content in tubers is higher than other metals [33].

The bioavailability and toxicity of metals in soil are significantly influenced by pH conditions. Soil pH is considered to be one of the most important factors that influence the transfer of metals from soil to plants, and higher pH values have been found to reduce the bioavailability and toxicity of some metals (Cd and Pb) [30,34,35]. The mechanism for this phenomenon can contribute to an increase in solubility and ion competition. As soil pH decreases, concentrations of Fe^2+^, Mn^2+^, Zn^2+^, and Ca^2+^ increases in soil solution. This enhances the competition of free ions and reduces the adsorption to soil particles [35]. Additionally, soil pH is the greatest determinant of the solubility and mobility of metals such as Cr, Pb, and Zn in sandy soil [36]. Due to the close relation between soil pH and heavy metal properties, correlation analysis of pH values and heavy metal accumulation is commonly used in research on bioconcentration of heavy metal from soil to crops [37]. 

#### 2.5.2. Foodstuff Consumption-Associated Health Risk Assessment

Potential human health risks of toxic metal exposure to residents of Tarkwa, through consumption of foodstuff were assessed using target hazard quotient (THQ) [38]. THQ is defined as the ratio of the body intake dose of a pollutant to the reference dose. If THQ > 1, there could be potential health risk associated with the pollutant. On the other hand, if THQ < 1, then, there will be no obvious risk. The THQ was calculated using the equation:

$$THQ=\frac{EFr\times ED\times FIR\times MC}{RfD\times BW\times AT}\;\times 10^{-3}$$

where MC is the geometric mean (GM) concentration of a particular metal in foodstuff (mg/kg ww), FIR is the daily intake by residents of Ghana (cassava: 601 and 400 g/day for adults and children respectively; plantain: 369 and 200 g/day for adults and children respectively) [39]. Daily intake of cassava and plantain by children were assumed. EFr is the exposure frequency (365 d/yr) [38], ED is the exposure duration (60 years for adults and 10 years for children) [40], BW is the average body weight of local residents (60 kg for adults and 32.7 kg for children) [6,9], AT is the average exposure time for non-carcinogens (21,900 and 3295 days for adults and children respectively), and RfD is the reference dose (RfD for As, Cd, Hg and Pb were 3E–04, 1E–03, 3E–04 and 3.5E–03 mg/kg/d respectively) [38].

#### 2.5.3. Statistical Analysis

Statistical analyses were performed using IBM SPSS 20.0 statistical software (SPSS Inc., Chicago, IL, USA) after data were normalized by log transformation. ANOVA and Tukey analyses were used to compare concentrations of metals in soil, cassava, and plantain (mg/kg dw), and differences were considered statistically significant with *p* value ˂ 0.05. Concentrations of heavy metals or metalloid below their respective LODs were replaced with a value of LOD/2. In this study, GM concentrations were used to represent the central tendency of heavy metals and metalloid in the study area [41]. The relationships between BCF of heavy metals in foodstuffs, soil pH, and SOM were examined by Pearson’s correlation and were considered statistically significant if *p* value was less than 0.05. Principal component analysis (PCA) based on log transformed data was done, to determine the distribution pattern of metals in foodstuffs. PCA was done using JMP statistical software v. 10 (SAS Institute). The principal components were extracted with eigenvalues > 1 through varimax rotation.

## 3. Results and Discussion

### 3.1. Heavy Metal Distribution in Agricultural Soils and Foodstuffs

Total concentrations of As, Cd, Co, Cr, Cu, Hg, Ni, Pb, and Zn in agricultural soils showed large variations in Tarkwa (Table 1; [29]). The GM concentrations of metals from the thirteen communities decreased in the order, Zn (37) > Cr (16) > Cu (5.1) > Pb (4.8) > As (3.8) > Ni (2.2) > Co (1.3) > Hg (0.49) and Cd (0.026) mg/kg dw (Table 1). Compared with ATSDR standard values [42], the mean concentrations of Hg in soil exceeded the limit (1 mg/kg) in Badukrom and Wangarakrom. Regarding regional distribution of metals in soil, concentrations of As and Hg in Badukrom and Wangarakrom were relatively higher than those in the other sampling communities around Tarkwa.

**Figure 2:**
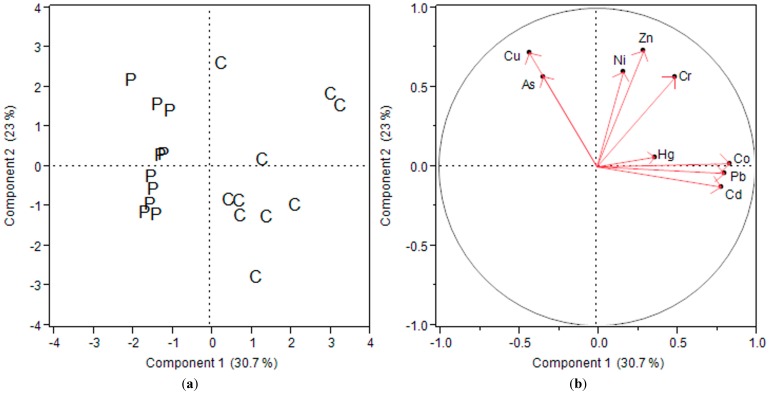
Distribution patterns of metals in foodstuffs characterized by PCA (C: Cassava; P: Plantain).

As compared to soils, foodstuffs contained significantly lower concentrations of heavy metals (*p* ˂ 0.01; Table 1) with obvious variations among the different species (Figure 2a). The GM concentration of metals in cassava decreased in the order: Zn (7.6) > Ni (3.7) > Cu (2.1) > Pb (0.18) > Cr (0.050) > Co (0.024) > As (0.0090) > Cd (0.0070) and Hg (0.0040) mg/kg ww (Table 1). 

Similarly, GM concentrations of metals in plantain decreased in the order, Zn (7.3) > Ni (3.6) > Cu (3.4) > Cr (0.025) > As (0.012) > Pb (0.0070) > Co (0.0054) > Cd (0.0010) and Hg (0.0010) mg/kg ww (Table 1). 

As shown in Figure 2, As and Cu were highly distributed in plantain while Cd, Co, Cr, Hg, Pb, Ni, and Zn were distributed in cassava. This trend in metal distribution could be due to the fact that cassava is a root tuber, and accumulation of metals in the edible part from contaminated soil was highly possible due to the mining operations within the study area. However, the higher distribution of As in plantain could be due to atmospheric deposition. Processing of the ore involves roasting, which results in the production of arsenic trioxide gas which is distributed throughout the study area by the wind [7].

**Table 1:** Concentrations (geometric mean, SD, minimum, maximum) of heavy metals and metalloid in soil (mg/kg dw) and foodstuffs (mg/kg ww) in Tarkwa.

Sample Type	Sample Number		As	Cd	Co	Cr	Cu	Hg	Ni	Pb	Zn
Soil ^#^	60	Geometric Mean	3.8 **^a^**	0.026 **^a^**	1.3 **^a^**	16 **^a^**	5.1 **^a^**	0.49 **^a^**	2.2 **^a^**	4.8 **^a^**	37 **^a^**
		SD	3.4	0.013	0.74	10	1.9	0.85	1.0	3.5	24
		Median	2.9	0.022	1.2	12	5.5	0.13	1.9	3.8	32
		Minimum	1.0	0.010	0.37	8.1	2.8	0.030	1.1	1.6	9.8
		Maximum	13	0.052	3.0	38	8.9	2.4	4.5	13	86
											
Cassava	33	Geometric Mean	0.0090 **^b^**	0.0070 **^b^**	0.024 **^b^**	0.050 **^b^**	2.1 **^b^**	0.0040 **^a^**	3.7 **^b^**	0.18 **^b^**	7.6 **^b^**
		SD	0.0050	0.0050	0.013	0.054	0.83	0.0030	0.68	0.17	2.2
		Median	0.0070	0.0050	0.024	0.038	2.3	0.0030	3.6	0.14	6.8
		Minimum	0.0030	0.0020	0.010	0.010	1.0	0.0010	2.7	0.017	4.7
		Maximum	0.017	0.016	0.051	0.20	3.3	0.010	5.4	0.45	12
											
Plantain	32	Geometric Mean	0.012 **^b^**	0.0010 **^b^**	0.0054 **^b^**	0.025 **^b^**	3.4 **^b^**	0.0010 **^a^**	3.6 **^b^**	0.0070 **^b^**	7.3 **^b^**
		SD	0.0060	0.0010	0.0030	0.014	1.0	0.0010	0.33	0.0060	1.9
		Median	0.011	0.0010	0.0050	0.023	3.4	0.0010	3.7	0.0050	6.6
		Minimum	0.0060	nd	0.0017	0.0090	1.9	0.0010	2.9	0.0022	4.8
		Maximum	0.026	0.0020	0.011	0.056	5.1	0.0045	4.1	0.020	10
											
ANOVA *p* value			˂0.01	˂0.01	˂0.01	˂0.01	˂0.01	≤0.05	˂0.01	˂0.01	˂0.01

nd: not detected; Different letters (^a^ and ^b^) between samples indicates significant difference (Tukey’s test; *p* ˂ 0.05); ^#^: Data for heavy metals in agricultural soils could be obtained from Bortey-Sam *et al*. [29].

Of the cassava samples collected, 30% contained higher concentration of Pb (0.37–0.45 mg/kg ww) when compared to the Codex Alimentarius Commission (CAC) standard values (0.3 mg/kg). However, concentrations of As, Cd, Cr, and Hg in all samples were below the CAC [43] standard values (0.05, 0.2, 0.5 and 0.01 mg/kg, respectively). Regarding regional distribution, cassava samples with high concentrations of Pb were mainly from Techiman, Wangarakrom, Samahu, and Tebe (Table 3). Among common pollutants that affect plants, Pb is one of the most toxic and frequently encountered [44]. Pb continues to be widely used in many industrial processes and occurs as a contaminant in all environmental compartments (soils, water, the atmosphere, and living organisms). Environmental Pb contamination results both from its persistence and from its present and past numerous sources. These sources may include smelting, combustion of leaded gasoline, or fertilizers application [44,45,46].

Previous study on the levels of toxic metals in *Xanthosoma sagititolium* (cocoyam) and *Colocasia esculenta* (water cocoyam), in Tarkwa showed higher concentrations than in this study. From the results of their study, the levels of As, Cd, and Hg in cocoyam and water cocoyam were higher than those of the WHO recommended levels [26]. These root tubers absorb toxic chemicals from soil as a result of the mining operations [26]. Similarly, Hayford *et al*. [25] reported that, concentrations of heavy metals in cassava and plantain were higher than values proposed by the FAO, Expert Committee on Food Additives of the WHO and ATSDR in mining communities around the Tarkwa–Prestea area. The differences in concentrations between this study and the study by Hayford *et al*. [25] could be due to fact that the uptake and allocation of metals in plants is influenced by life history, traits, climate, stage, age, nutritional status, disease, and rhizosphere interactions with micro-organisms [19]. Another possible reason could be due to differences in sample points.

### 3.2. Bioaccumulation of Heavy Metals in Foodstuffs from Soil 

Bioconcentration of heavy metals and metalloid from soil to plants depend on soil characteristics, metal chemistry and also on the plant species. BCF values of the metals studied in different foodstuffs were calculated (on dry weight basis) and data are provided in Table 2. 

In all the foodstuffs tested, the mean BCF of Ni (2.0 ± 0.87 (cassava) and 2.2 ± 0.96 (plantain)) was highest and that of Cr was lowest (0.0035 ± 0.0036 (cassava) and 0.0021 ± 0.0011 (plantain)). As shown in Table 1, concentrations of Ni in soil and foodstuff differed considerably (*p* ˂ 0.05) and levels in foodstuffs were significantly higher compared to soil (*p* ˂ 0.05). The average BCF of metals and metalloid in the different foodstuffs are shown in Table 2.

Since a greater BCF value indicates a higher transfer potential of metals in vegetables/foodstuffs [47], the above results indicated that Ni had higher capacity of absorption into food crops from soil than the other heavy metals studied. The study further showed that there was no significant correlation between SOM, soil pH, and BCF values of heavy metals in the foodstuffs (*p* > 0.05). Thus soil pH and SOM had minor or no significant effect on the accumulation of metals in foodstuffs. This study is consistent with other study [48] which indicated that pH had no significant effect on the accumulation of metals (Pb) from soil to vegetables.

**Table 2:** Average soil pH, organic matter, water contents, and bioconcentration factors (BCF) of heavy metals and metalloid in foodstuffs from agricultural soils in Tarkwa.

	Sample Site	n	Soil pH	SOM	WC	As	Cd	Co	Cr	Cu	Hg	Ni	Pb	Zn
C	Mile 7	3	7.3	1.8	0.91	0.0058	1.1	0.019	0.004	0.64	0.067	3.2	0.13	0.70
	Mile 8	3	7.1	1.8	0.88	0.0043	0.25	0.035	0.001	0.49	0.021	2.3	0.020	0.34
	Techiman	3	6.3	1.8	0.66	0.0034	0.24	0.005	0.001	0.19	0.015	1.1	0.16	0.15
	Wangarakrom	3	6.9	2.8	0.66	0.0031	0.57	0.018	0.013	0.68	0.002	1.1	0.15	0.45
	Badukrom	3	6.9	2.0	2.5	0.00034	1.2	0.11	0.001	0.55	0.001	2.8	0.055	0.22
	Samahu	3	7.1	2.4	2.1	0.0016	0.24	0.041	0.002	0.45	0.020	1.8	0.049	0.34
	Abekuase	3	7.0	2.5	2.1	0.0048	0.14	0.009	0.007	0.87	0.15	2.9	0.013	0.22
	Tebe	3	6.9	2.9	1.9	0.0038	0.39	0.013	0.003	0.41	0.070	1.6	0.073	0.55
	Huniso	3	7.3	1.5	0.99	0.010	0.20	0.031	0.004	0.36	0.006	2.8	0.001	0.073
	Pepesa	3	7.3	1.9	1.9	0.0011	0.041	0.036	0.002	0.38	0.049	1.8	0.004	0.083
	Teberebe	3	7.6	2.5	1.1	0.0036	0.11	0.0035	0.001	0.11	0.023	0.59	0.003	0.14
	Average		7.1	2.2	1.4	0.004	0.40	0.029	0.0035	0.47	0.039	2.0	0.060	0.29
	SD		0.34	0.46	0.67	0.003	0.39	0.030	0.0036	0.22	0.045	0.87	0.061	0.20
	Minimum		6.3	1.5	0.66	0.00034	0.041	0.0035	0.0010	0.11	0.0014	0.60	0.0012	0.07
	Maximum		7.6	2.9	2.5	0.010	1.2	0.11	0.013	0.87	0.15	3.2	0.16	0.70
P	Mile 7	3	7.3	1.8	0.91	0.0085	0.12	0.0062	0.0024	1.3	0.033	3.7	0.0052	1.1
	Mile 9	3	5.8	2.1	0.50	0.0030	0.011	0.0043	0.00057	0.91	0.0061	2.3	0.0011	0.49
	Mile 10	3	7.2	1.3	0.44	0.0093	0.024	0.0068	0.00073	1.5	0.023	3.8	0.0012	0.21
	Techiman	3	6.3	1.8	0.66	0.0075	0.031	0.0044	0.0023	0.93	0.0053	1.2	0.0018	0.32
	Wangarakrom	4	6.9	2.8	0.66	0.0026	0.070	0.0024	0.0022	0.99	0.0023	1.1	0.0060	0.35
	Badukrom	3	6.9	2.0	2.5	0.00093	0.011	0.015	0.0014	0.39	0.00041	2.8	0.00096	0.17
	Abekuase	3	7.0	2.5	2.1	0.0035	0.024	0.0060	0.0031	0.89	0.018	1.9	0.0019	0.21
	Tebe	3	6.9	2.9	1.9	0.0092	0.13	0.0024	0.0044	0.84	0.014	1.6	0.0048	0.46
	Huniso	3	7.3	1.5	0.99	0.0077	0.005	0.015	0.0020	0.73	0.0054	2.5	0.00031	0.074
	Pepesa	4	7.3	1.9	1.9	0.0033	0.030	0.0020	0.0012	0.28	0.0040	1.5	0.00053	0.082
	Average		6.9	2.1	1.3	0.0056	0.045	0.0065	0.0021	0.88	0.011	2.2	0.0024	0.35
	SD		0.49	0.53	0.76	0.0032	0.044	0.005	0.0011	0.37	0.011	0.96	0.0021	0.29
	Minimum		5.8	1.3	0.44	0.00093	0.0051	0.0020	0.00057	0.28	0.00041	1.1	0.00031	0.07
	Maximum		7.3	2.9	2.5	0.0093	0.13	0.015	0.0044	1.4	0.033	3.8	0.0060	1.1

C: Cassava; P: Plantain; n: number of samples collected: SOM; Soil organic matter (%); WC: Soil water content (%).

**Table 3:** Target hazard quotients of toxic metals via foodstuff consumption in Tarkwa.

		Adult				Child			
	Sample Site	As	Cd	Hg	Pb	As	Cd	Hg	Pb
C	Mile 7	0.21	0.13	0.07	0.60	0.28	0.17	0.090	0.81
	Mile 8	0.19	0.05	0.13	0.13	0.26	0.07	0.18	0.18
	Techiman	0.12	0.05	0.09	**1.3**	0.16	0.07	0.12	**1.7**
	Wangarakrom	0.57	0.12	0.11	**1.1**	0.77	0.17	0.14	**1.4**
	Badukrom	0.15	0.16	0.12	0.39	0.20	0.22	0.16	0.52
	Samahu	0.25	0.06	0.07	**1.2**	0.34	0.08	0.10	**1.6**
	Abekuase	0.52	0.03	0.25	0.14	0.71	0.05	0.34	0.19
	Tebe	0.37	0.04	0.12	0.86	0.50	0.05	0.16	**1.2**
	Huniso	0.50	0.10	0.03	0.05	0.67	0.14	0.036	0.064
	Pepesa	0.19	0.02	0.32	0.06	0.26	0.02	0.44	0.079
	Teberebe	0.33	0.04	0.05	0.05	0.44	0.06	0.07	0.066
P	Mile 7	0.19	0.0086	0.021	0.014	0.21	0.0095	0.023	0.015
	Mile 9	0.11	0.00082	0.016	0.0039	0.13	0.00090	0.018	0.0043
	Mile 10	0.18	0.0029	0.014	0.0045	0.20	0.0032	0.016	0.0049
	Techiman	0.16	0.0039	0.018	0.0091	0.18	0.0042	0.020	0.010
	Wangarakrom	0.29	0.0093	0.092	0.027	0.32	0.010	0.10	0.029
	Badukrom	0.25	0.00091	0.021	0.0042	0.27	0.0010	0.022	0.0045
	Abekuase	0.23	0.0036	0.018	0.013	0.26	0.0039	0.020	0.014
	Tebe	0.54	0.0080	0.014	0.035	0.60	0.0087	0.015	0.038
	Huniso	0.24	0.0016	0.014	0.0074	0.26	0.0018	0.016	0.0082
	Pepesa	0.33	0.0076	0.016	0.0052	0.36	0.0084	0.018	0.0057

C: Cassava; P: Plantain; THQ: Target hazard quotient; Bold indicates high THQ.

### 3.3. Potential Human Health Risk Associated with Consumption of Foodstuffs

The potential human health risk of As, Cd, Hg, and Pb to local residents associated with cassava and plantain consumption was assessed using THQ index and data are shown in Table 3. For both children and adults, the THQs of Pb in cassava in Techiman, Wangarakrom, Samahu, and Tebe (only children) were greater than 1.0 (Table 3). This meant that residents could be exposed to significant health risks associated with cassava consumption. Children were especially vulnerable to acute, sub–acute and chronic effects of ingestion of chemical pollutants, since they (children) consumed more (twice of the amount) of food per unit of body weight compared to adults [49]. On the other hand, the THQs of As, Cd, Hg, and Pb in plantain were less than 1. The high THQs found in cassava, and especially Pb, could be due to the greater consumption rate, because human health risk assessment does not only depend on the toxic metal concentration in food, but also on the consumption rate [50].

The people of Ghana including the residents living in the study area of this report, generally had high requirement for cassava. The daily intakes of cassava and plantain were at an average of 601 g/day and 369 g/day respectively [43] and prolonged consumption of these toxic metals could lead to accumulation and cause toxicity, and therefore there was a clear need for proper treatment before consumption. Furthermore, continuous monitoring of As, Cd, Hg, and Pb residues (in foodstuffs, vegetables, fruits, *etc*.) in the vicinities of gold mines in Tarkwa is recommended in the interest of consumers.

## 4. Conclusions

The GM concentration of heavy metals and metalloid in foodstuffs collected from communities in Tarkwa decreased in the order: Zn > Ni > Cu > Pb > Cr > Co > As > Cd and Hg (cassava) and, Zn > Ni > Cu > Cr > As > Pb > Co > Cd and Hg (plantain) respectively. The transfer of these heavy metals and metalloid in foodstuffs from soil was assessed and bioconcentration factor indicated that Ni had higher capacity of absorption into food crops from soil than the other metals studied in Tarkwa. THQ of Pb showed that there could be potential health risk to residents (Techiman, Wangarakrom, Samahu and Tebe) through consumption of cassava. Children were especially vulnerable to acute, sub–acute, and chronic effects of ingestion of chemical pollutants, since they (children) consumed more food (twice of the amount) per unit of body weight compared to adults [49]. 
